# Applications and Advances in Bioelectronic Noses for Odour Sensing

**DOI:** 10.3390/s18010103

**Published:** 2018-01-01

**Authors:** Tran Thi Dung, Yunkwang Oh, Seon-Jin Choi, Il-Doo Kim, Min-Kyu Oh, Moonil Kim

**Affiliations:** 1 Hazards Monitoring Bionano Research Center (HMBRC), Korea Research Institute of Bioscience and Biotechnology (KRIBB), 125 Gwahak-Ro, Yuseong-Gu, Daejeon 34141, Korea; dungtran6386@gmail.com (T.T.D.); oyk0213@kribb.re.kr (Y.O.); 2 Department of Nanobiotechnology, Korea University of Science and Technology (UST), 217 Gajeong-Ro, Yuseong-Gu, Daejeon 34113, Korea; 3 Department of Chemical and Biological Engineering, Korea University, 145 Anam-Ro, Sungbuk-Gu, Seoul 02841, Korea; 4 Department of Chemistry, Massachusetts Institute of Technology, 77 Massachusetts Avenue, Cambridge, MA 02139, USA; sjchoi27@kaist.ac.kr or seonjin@mit.edu; 5 Department of Materials Science and Engineering, Korea Advanced Institute of Science and Technology (KAIST), 291 Daehak-Ro, Yuseong-Gu, Daejeon 34141, Korea; idkim@kaist.ac.kr; 6 Department of Pathobiology, College of Veterinary Medicine Nursing & Allied Health (CVMNAH), Tuskegee University, Tuskegee, AL 36088, USA

**Keywords:** bioelectronic nose, olfactory receptor, OR, odour analysis, biosensor

## Abstract

A bioelectronic nose, an intelligent chemical sensor array system coupled with bio-receptors to identify gases and vapours, resembles mammalian olfaction by which many vertebrates can sniff out volatile organic compounds (VOCs) sensitively and specifically even at very low concentrations. Olfaction is undertaken by the olfactory system, which detects odorants that are inhaled through the nose where they come into contact with the olfactory epithelium containing olfactory receptors (ORs). Because of its ability to mimic biological olfaction, a bio-inspired electronic nose has been used to detect a variety of important compounds in complex environments. Recently, biosensor systems have been introduced that combine nanoelectronic technology and olfactory receptors themselves as a source of capturing elements for biosensing. In this article, we will present the latest advances in bioelectronic nose technology mimicking the olfactory system, including biological recognition elements, emerging detection systems, production and immobilization of sensing elements on sensor surface, and applications of bioelectronic noses. Furthermore, current research trends and future challenges in this field will be discussed.

## 1. Introduction

The possibility of the use of electronic instruments to measure odour intensity were examined in the early 1960s [1], but the modern artificial olfactory system was first built in 1982 by Persaud and Dodd who used a microsensor gas array based on metal-oxide structure to identify odours [2]. The name “electronic nose”, however, appeared for the first time in 1987, and the current definition was given by Gardner in 1988 [3]. In most electronic nose systems, sensor arrays are adopted to test volatile molecules in the gas phases. When the sensors come in contact with volatile organic compounds (VOCs), the sensor surface undergoes a physical or chemical change of the sensor [4], and generally its resultant signals are converted into digital values. The commonly used sensors include surface plasmon resonance (SPR) [5], quartz crystal microbalance (QCM) [6], surface acoustic wave (SAW) [7], bulk acoustic wave (BAW) [8], conducting polymers (CP) [9], field-effect transistor (FET)-type transducers [10], etc. Although electronic noses are sensitive to odorants in a specific way, most electronic noses face a significant challenge in terms of specificity of the sensors. This new concept for chemical sensor is referred to as a “bioelectronic nose”, which can detect specific odours with high selectivity. The bioelectronic nose has a limit of detection (LOD) at the fM levels in liquid solutions and ppt levels in gaseous conditions, which is similar to that of the human sense of smell [11,12].

In recent years, in order to overcome the drawbacks of electronic noses, many artificial olfactory sensors based on biomaterials such as mammalian cells or olfactory receptors have been developed [13,14]. Especially, olfactory receptor (OR)-based gas sensors designed to mimic the olfactory system have been considered among the most promising tools for detection of various odorants with high sensitivity and selectivity. The bio-inspired electronic noses utilizes biological ORs or cells expressing ORs as recognition elements, together with sensor devices, which produce and amplify electrical signals from the biological interaction of odorant molecules with their ORs. Accordingly, the distinct merit of the bioelectronic nose, as opposed to a conventional electronic nose, is its ability to perform high sensitive and specific measurement of target odorants.

The biological olfaction system has the ability to detect and discriminate thousands of low molecular weight compounds with various chemical structures and properties. In the olfactory system, ORs play a critical role in chemosensory signal transduction. Animals ranging from nematodes to humans sense their chemical environments through ORs [15]. Recent experiments have ascertained that ORs alone, even when expressed in heterologous systems, can be activated to transduce the signaling cascade [16]. In 1991, Nobel Laureates in Physiology or Medicine Buck and Axel carried out a series of pioneer studies that clarified how our olfactory system works [17]. ORs belong to the G protein-coupled receptors (GPCRs), which are a very large family of transmembrane receptors with seven transmembrane helices that recognize a number of odorant compounds with high selectivity, and trigger signal transduction in olfactory neurons. The mammalian olfactory system has the ability to detect thousands of volatile molecules at very low concentrations and even to discriminate between some of them that differ by only one or a few atomic mass units [18]. Due to their specificity for odours and biomimetic properties, ORs have been adopted to detect a target molecule from all the compounds in a mixture [19,20,21]. ORs as recognition elements have some of the benefits compared to olfactory tissue and cells, such as longer-term stability, higher level of activity, and much easier maintenance. Since the first proof-of-concept study using bioelectronic nose by Gopel et al. in 1998 [22], a variety of OR-based biosensors have been studies intensively over the past two decades, which employed the extracted membrane proteins containing expressed ORs or partially purified ORs as the sensitive materials [23,24].

**Figure 1:**
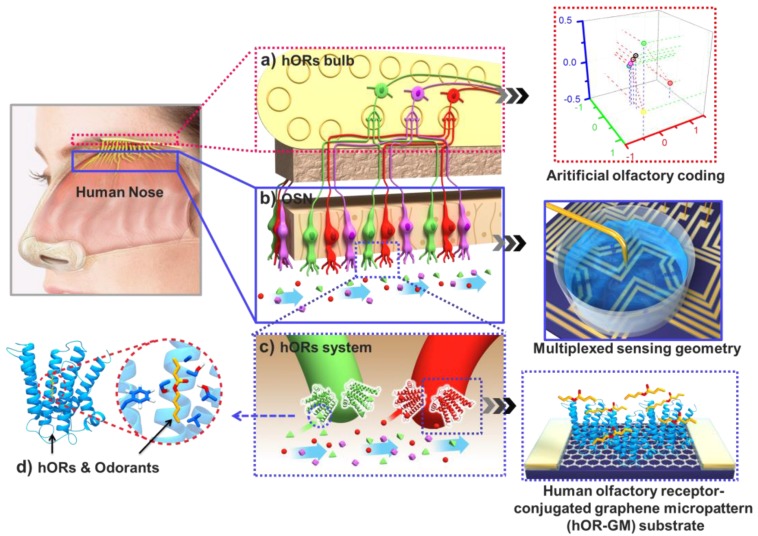
Schematic diagram of functional anatomy of human olfactory system and components of bioelectronic nose [13]. (**a**) Olfactory bulb, where the olfactory signals generated by OSNs are combined for the generation of combinatorial olfactory codes, matching with artificial olfactory codes generated by MSB-nose. (**b**) OSNs, where olfactory signals triggered by the specific binding of hORs and odorants, matching with GMs functionalized with hORs. (**c**) hORs for the specific recognition of odorants. (**d**) Illumination of specific interaction between hOR and odorant.

A bioelectronic nose is schematically compared with a human olfactory system [13]. Figure 1 shows a basic anatomy of the human olfactory system and functional relationship within each stage between bioelectronic nose and human olfaction. In Figure 1a–c, when odorants are exposed to the nasal cavity, they are selectively recognized by ORs, which triggers intracellular signal transduction cascades and induces the depolarization of olfactory sensory neurons (OSNs). The chemical information of odorants is converted into the electric signal of OSNs, and transmitted via the olfactory bulb into the brain cortex for processing. Figure 1d shows structural features of the odorant binding site of human olfactory receptor. Hypervariable sites of amino acids in the transmembrane helices of ORs are located near a binding pocket for a specific odorant molecule. These receptors are located on the olfactory receptor cells, which occupy a small area in the upper part of the nasal epithelium and detect inhaled odorant molecules. When ORs are activated by the odorants, an electric signal is triggered in olfactory receptor cells and sent to the brain via nerve processes. Recently, a central repository of olfactory receptor and olfactory receptor-like gene and protein sequences have been organized and stored at the Olfactory Receptor Database (ORDB) (http://senselab.med.yale.edu/ordb) by Yale University School of Medicine. This database provides useful information on the gene expression patterns and the integrative properties of neurons [25]. Currently, olfactory research is focused on the discovery of potential commercial applications, and the biomimetic design of an electronic nose is considered a significant breakthrough [26]. Thanks to advances in nanotechnology, receptor proteins have been applied to different types of transducers, such as QCM [27,28], SPR [21,29] and FET [13,30,31].

In this article, we will focus on the most recent advances in the development of biomimetic artificial noses, including whole cell, olfactory receptor protein and odorant binding protein (OBP)-based biosensors. Three important issues in this field are biological recognition elements, immobilization methods and sensor formats. This review will focus mainly on their working principles, performance, merits and drawbacks. The newest advances and applications will be summarized and future challenges will be discussed. A brief history of the bioelectronic noses is shown in Table 1.

## 2. Biological Recognition Elements

Olfactory receptor neurons express olfactory receptors on the cell membranes. The activated ORs are the initial process in a signal transduction cascade, which produces action potentials (or nerve impulses) in neurons that eventually reach the brain. ORs have a binding affinity for a range of odour molecules rather than specific binding of particular ligands, and conversely, each odour molecule may bind to several receptors with overlapping ligand affinities [36]. ORs could be applied in sensor systems as whole cell expressing ORs, ORs located on a membrane fraction or nanovesicles and OBPs themselves.

### 2.1. The Use of Whole Cell Expressing Olfactory Receptors in Bioelectronic Nose

For the engineering of olfactory receptor proteins, *Escherichia coli*, *Saccharomyces cerevisiae* and human embryonic kidney (HEK) cells are most widely employed as olfactory-receptor-carrying cells to produce recognition elements in bioelectronic noses [37]. In an early study on whole cell sensing using bioelectronic noses, Wu developed a piezoelectric electrode used in the immobilization of a crude bullfrog cilia as a signal transducer in 1999 [27]. In that study, trace levels of various odorants were detected at various concentrations highly correlated with the olfactory threshold values of the human nose using the piezoelectric biosensor. Another study on whole cell biosensor based on a yeast expression system was reported to identify mutations within residues of estrogen receptor-α (ERα) responsible for ligand binding and mutations that influence protein activity or expression [38]. Intracellular binding of small molecule ligands to proteins resulted in changes in growth of temperature-sensitive yeast. Estrogen analogs could be distinguished using the ERα sensor by detecting differences in growth rates of yeast that positively correlated with relative affinities of the analogs for binding to the ERα. The ERα sensor system provided an easy-to-use and cost-effective assay, and might be useful for screening for novel ligands and ligand-binding domains. Fukutani et al. developed a new type of a yeast-based biomimetic odour sensor [39]. In that study, the replacement of the N-terminal region of the mouse olfactory receptor OR226 with the corresponding regions of the rat I7 receptor mOR226 affected the expression and localization of the receptor and improved the sensing ability of the yeast cells for 2,4-dinitrotoluene (DNT). Their strategy has potential for establishment of an odour sensor system with OR-expressing yeast, elevating the odorant-sensing ability of the yeast cells. Lee et al. employed SPR system to characterize molecular interactions between olfactory receptors and their cognate odour molecules [29]. The SPR system was applied to the cell-based measurement of odorants, in which HEK-293 cells were used as a heterologous cell expression system, and *Caenorhabditis elegans* olfactory receptor ODR-10 which is capable of detecting diacetyl was adopted as a model olfactory receptor. The SPR signals were obtained from HEK-293 cells expressing ODR-10 after exposure to 0.1 mM diacetyl, while no signal was observed from control HEK-293 cells not expressing ODR-10. The results demonstrated that the SPR system coupled with a heterologous olfactory system could be used to detect odorants specific to each odour receptor molecule. A whole cell-based QCM sensor system for selective recognition of odorant molecules was developed by Ko and Park [32]. The signals obtained from QCM coated with HEK-293 cells containing the olfactory receptor rat I7 indicated that its specific odorant, ocetyl aldehyde, interacted with the expressed I7 receptor, which could be quantitatively measured. The use of whole cells expressing olfactory receptors as recognizing elements of electronic noses has been reported over the last thirty five years. To date, bacterial cells have been most extensively used for whole cell-based detection as biological sensing element. Bacterial cell-based sensing systems have a major problem that they may lack robustness and suffer from short shelf life or in-use life required for commercial application of whole cell sensors. Yeast cells, however, are more stable and durable than many bacterial cells and may make it possible to overcome the drawback associated with bacterial cell-based sensors [38,40]. A further distinct advantage of the use of yeasts, the simplest eukaryotes, is that they can be used to give information more directly applicable to plant and animal and, because many essential cellular processes are similar between yeast and these eukaryotic organisms [41].

### 2.2. The Use of Olfactory Receptor Proteins in Bioelectronic Nose

The use of isolated olfactory receptors instead of whole cells makes it possible to scale down biosensors, making them more applicable to nanotechnology. Immobilization of receptors onto a sensor solid surface in a manner to preserve their functional activities is of great importance in developing a bioelectronic nose. As mentioned in the introduction, olfactory receptors are extremely hydrophobic, so it is difficult to functionally stabilize the receptors; therefore, using a heterologous cell membrane carrying an olfactory protein is a promising method. A crude membrane expressing an olfactory protein was used for measuring odorants using a QCM [28]. In that study, the surface was coated with a crude membrane expressing olfactory receptor protein ODR-10 extracted from *E. coli* then it examined its interaction with various odorant molecules, showing a liner dose-dependent response of the piezoelectronic biosensor upon membrane extraction with the natural receptor ligand diacetyl. Similar research by Segui et al., in the same year, used a membrane fraction carrying olfactory receptor protein and rat olfactory receptor I7 as a sensing element; the study provided the first step toward developing a QCM olfactory sensor [42]. For the sensor, a self-assembled multilayer composed of a mixed MHDA-biotinyl PE self-assembled monolayer and a biotin–avidin bridge system was grafted onto the sensor surface, and a receptor-specific biotinylated antibody was used to recognize a membrane fraction containing I7 receptor protein.

**Figure 2:**
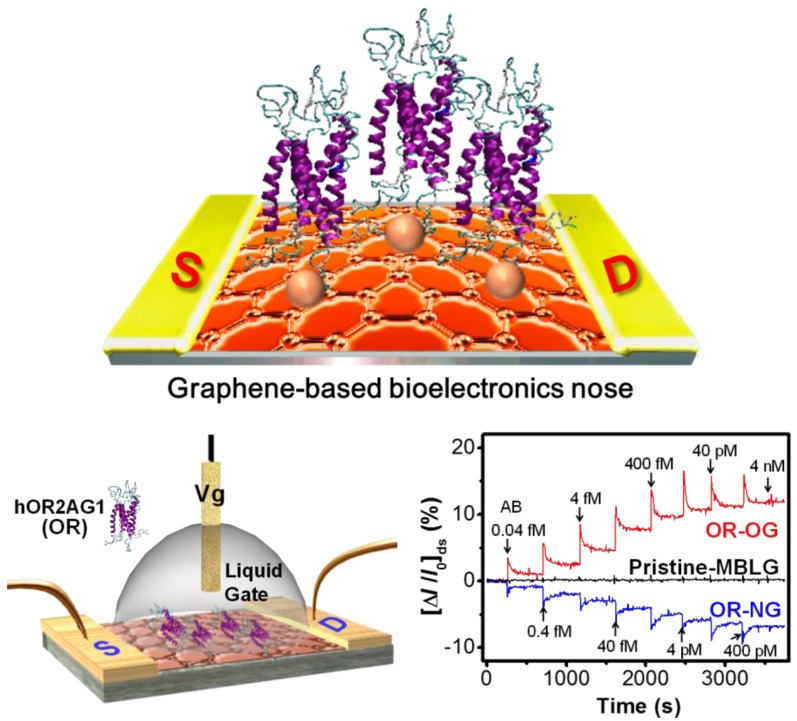
Schematic diagram of a liquid-ion-gated FET bioelectronic nose using OR-conjugated modified bilayer graphene (MBLG) [14].

Transmembrane proteins are insoluble and require a specific detergent environment to maintain their natural structure and native function [43]. Recently, Park et al. reported an ultrasensitive and flexible FET olfactory system [14]. As shown in Figure 2, the authors developed the FET-type bioelectronic nose based on the modified bilayer graphene (MBLG) integrated with the olfactory receptor hOR2AG1 for specific recognition of amyl butyrate (AB). In that study, the minimum detection limit (MDL) was as low as 0.04 fM, which was approximately two orders of magnitude more sensitive than previously reported olfactory sensors. In one study, nanotubes were integrated to a microelectronic array to create a FET, giving measurable signals down to femtomolar concentration levels of specific odorants [44]. The authors used the membrane fraction harboring human olfactory receptor 2AG1 (hOR2AG1) covalently attached by an amino-link to carboxylic acid-functionalized conducting polymer nanotubes.

**Figure 3:**
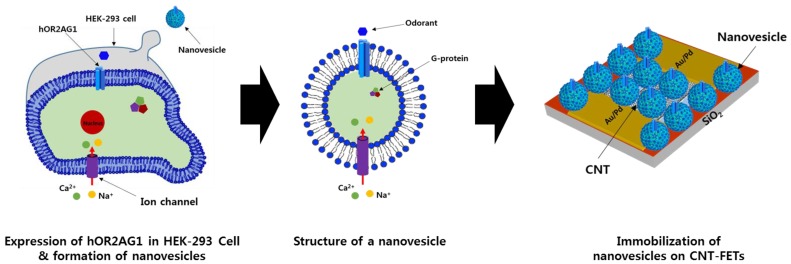
Schematic diagram depicting the preparation of nanovesicles containing hOR2AG1 and the immobilizaion of nanovesicles on SWNT-FET transducers. Adapted from [33].

In general, olfactory receptor proteins are expressed in heterologous cells, then solubilized in an adequate detergent before being integrated into nanovesicles serving as a matrix for protein reconstitution [45]. The artificial nanovesicle-based bioelectronic nose system mimicking human nose responses to odorant molecules was first introduced in 2012 by Jin et al. [33] and has been intensively and extensively developed in odorant recognition with high selectivity and sensitivity (Figure 3). Briefly, HEK-293 cells are transiently transfected with hOR2AG1-expressing construct, and then nanovesicles are produced from the hOR2AG1-expressed HEK-293 cells and separated from the cells. The nanovesicles retain membrane proteins and cytosolic components, ensuring the partial imitation of the hOR protein-mediated signal transmission. Then, the fabricated SWNT-FETs are incubated in the nanovesicle solution to form a nanovesicle-based bioelectronic nose. This platform has been applied in various fields, especially in medical diagnosis and food quality control [46,47]. In general, the bioelectronic nose system without nanovesicles measures changes in charge state of the receptor molecules, upon binding of odorants to their receptors. In contrast, in nanovesicle-based bioelectronic noses, interaction between odorants and ORs triggers cell signal pathways, and leads to a charge accumulation in the nanovesicles, thereby allowing sensitivity amplification in signal transduction [33]. Effort to interface nanoelectronic devices to olfactory receptor proteins was carried out by Goldsmith et al. in 2011 [19]. They integrated olfactory receptors with CNT transistors to detect molecules outside eukaryotic cells in the gas phase under ambient condition. In that study, mouse olfactory receptors (mORs) were employed and purified from cells then solubilized in digitonin, a surfactant containing a cholesterol-like backbone, or in nanodiscs, disk-shaped protein–lipid particles. The device responses substantially relied on mOR identity, odorant identity, and odorant concentration. In a recent study, Oh et al. detected odorants by using olfactory receptors in the SPR system [21]. After purification as an inclusion body, a human olfactory receptor hOR3A1 protein was purified and reconstituted using lipid/detergent-mixed micelles to form proteoliposomes. The results demonstrated that reconstituted hOR3A1 detected its cognate odour helional in a dose-dependent manner and also discriminated it from other odorants including structurally similar odorants. Examples of OR-based bioelectronic noses are listed in Table 2.

### 2.3. The Use of OBPs in Bioelectronic Nose

**Figure 4:**
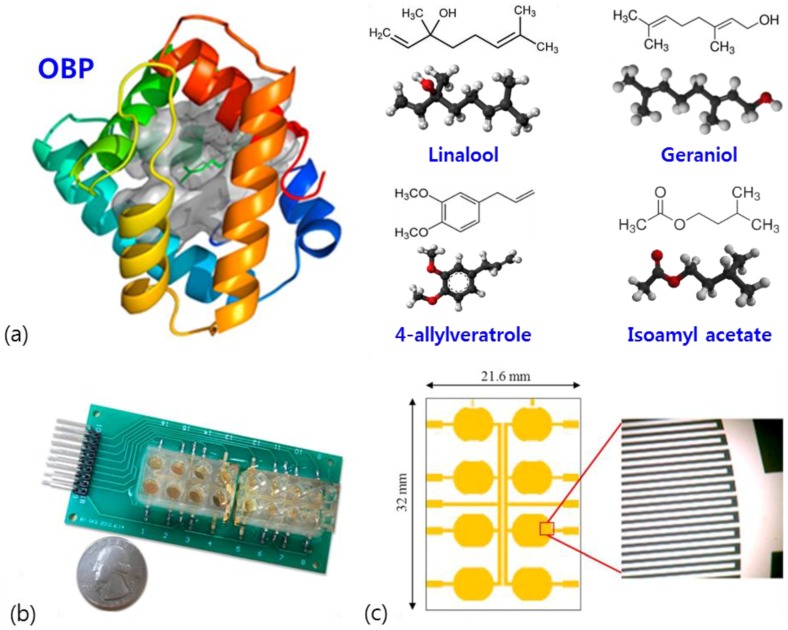
The structure of Acer-ASP2 and interdigitated electrodes for impedance detection. (**a**) Molecular structures of OBP and its four ligands, linalool, geraniol, 4-allylveratrole and isoamyl acetate. (**b**) Electrode device of the biosensor system. (**c**) Structure of the interdigitated electrodes on the bottom of a well. Adapted from [35].

OBPs are small water-soluble polypeptides found in the secretory glands as well as in the sensory organs of insects and vertebrates, and serve to recognize or release the stimuli of odorant molecules. In insects, the first step in the detection of odour compounds is the capture of the odorants by some extracellular proteins and membrane-bound ORs. One type of major peripheral olfactory proteins that recognize odour molecules is OBPs. Insect OBPs are expressed not only in olfactory tissues but also in non-olfactory tissues including gustatory sensilla and other specialized tissues, and serve as carrier proteins with a wide range of specificities for lipophilic compounds. The insect OBP is one of the most promising candidates in biointerface technology, which plays a critical role in improving bioelectronic nose performance for the monitoring of VOCs. The honeybee is an insect model that is useful for performing olfactory research and there has been remarkable progress in establishing its olfactory signaling mechanism through neurobiological and behavioral studies. Recently, the binding properties of an odorant-binding protein Acer-ASP2 from the honeybee to its ligands, the tertiary structure of the OBP and the protein–ligand interactions were investigated by molecular docking [35]. As shown in Figure 4, the honeybee Acer-ASP2 possessing good affinities with various ligands, such as floral odours and some pheromones, was immobilized on the surface of an interdigitated gold electrode. The authors focused on establishing an impedance biosensor system coupled with Acer-ASP2 to explore the binding properties of the OBP to its ligands. Based on molecular docking analysis, an impedance model was suggested to explain the correlations between changes of protein conformations and electrical impedance.

Another recent research using OBP in combination with interdigitated electrodes was used for insect semiochemical analysis. In that study, OBPs from an oriental fruit fly, *Bactrocera dorsalis*, were successfully expressed, purified and immobilized on interdigitated electrodes by a specially designed polyethylene glycol (PEG), SH–PEG–COOH, to detect semiochemicals [51]. They demonstrated that interactions between OBPs and various semiochemicals released from insect host plants such as isoamyl acetate, beta-ionone and benzaldehyde could be detected by the electrochemical sensing techniques coupled with the molecular docking analysis. They also claimed that electrochemical impedance biosensors based on insect OBPs have the possibility of being applied in many sensing applications such as pest control, military and healthcare. Besides insect OBPs, a biosensor array system composed of five SAW resonators coated with three types of OBPs including wild-type OBP from bovine (wtbOBP), a double-mutant of the OBP from bovine (dmbOBP), and a wild-type OBP from pig (wtbOBP) was used to detect the vapour phase of odorant molecules [20]. In their study, OBPs were deposited at high resolution on the active region of SAW resonators using laser-induced forward transfer (LIFT) to discriminate between octenol and carvone molecules, demonstrating that OBP-based SAW bioelectronic noses have the ability to distinguish octenol from carvone and are potentially useful for evaluating food contamination by fungi. The OBP-based bioelectronic nose technology provides a useful approach for chemical molecular sensing by successfully detecting ligands such as flower scents and insect pheromones, and for studying the interaction between these specialized olfactory proteins and odour molecules.

## 3. Production and Immobilization of ORs as Sensing Elements

In the development, fabrication and performance of OR-based bioelectronic noses, the sensitive elements (whole cells, ORs, OBPs), as well as their coupling to transducers, are the most critical aspects. Therefore, the production and immobilization of these sensing elements are both crucial issues. Various OR production methods and immobilization techniques will be discussed in this part.

### 3.1. Production of ORs

The activity of the functional OR immobilized on the sensing layer of the bioelectronic nose directly affects the performance of the biosensor, including the sensitivity, specificity, and stability of the sensor. Therefore, the production of functional ORs is one of the most important factors in order to develop OR-based biosensors. The successful production of sensing elements must satisfy the following requirements: ability to selectively recognize the target ligand by retaining the original active structure of the receptor and its inherent function, low production cost and long shelf life. Even though many studies have developed and reported many technical solutions to establish more appropriate methods for producing functional ORs, there is no single method that could meet all of the above requirements. The current methods for OR production are shown in Table 3.

As mentioned before in this review, three types of olfactory receptors have been used, including whole cell expressing ORs, ORs themselves and OBPs. In the case of whole cell-based biosensors, ORs present in living olfactory or sensory cells are the most convenient biomaterials because they can be used directly as sensing elements of target ligands without protein engineering efforts. In very early research by Wu, ORs from bullfrogs were coated onto a sensor array for detection of distinct VOCs [27]. Another research used insect olfactory receptor neurons (ORNs) in vitro to detect odorants by recording action potential response with a microelectrode [52]. Rat olfactory mucosa tissues also were used to develop a bioelectronic nose by Liu et al. [53,54]. The most important advantage of the methods used in their studies is that the naturally active tertiary structure of the OR is conserved and the intercellular connectivity is properly maintained so that target odorant molecules can be efficiently detected. However, the main disadvantages of this method are as follows. It is difficult to quantitatively and qualitatively secure a target ligand-specific OR. It is also costly and inefficient to selectively purify a particular type of OR from live olfactory and sensory neurons. Most of all, since living tissues and cells are used, it is necessary to provide a specific environment for the sensor recognition layer in order to maintain those functions, which is not suitable for commercial applications of the biosensor.

Due to these considerable drawbacks of using whole cells as capturing agents derived from living organs, tissues and neurons, heterologous cell systems with expressed olfactory receptors have been employed to obtain ORs. For whole cell fabrication, *E. coli*, *S. cerevisiae* and HEK cells are the three most commonly used expressing systems [12,55]. The ORs expressed and isolated by using these heterologous expression methods are immobilized onto the biosensor surfaces. In this approach, the target genes of specific ORs are inserted into expression vectors, which could be used to transfect expression cells, finally resulting in the high expression of ORs in the heterologous cells. For the production of ORs using animal model systems, the sensing elements are usually extracted from rat and mouse in the research of OR-based electronic nose [56]. By using HEK-293 cell system, the human OR (hORI7-4) [57] and zebra fish OR (OR131-2) [58] have been successfully expressed and the extracted membrane proteins carrying high expressed ORs then were immobilized to form bioelectronic nose systems. This method offers several advantages such as high expression of the OR on the cell plasma membrane and maintenance of the active tertiary structure. This also enables to graft the tags required for efficient OR immobilization on the sensor surface and facilitates molecular biological design to improve the chemical ligand specificity for its cognate OR. Nevertheless, besides these merits, this method also has some drawbacks: the expression of ORs in heterologous cells is labor-intensive, time-consuming and inefficient. Moreover, this method produces some irrelevant proteins besides the target protein, which requires additional specific purification. This approach looks similar to whole cell expression of ORs in heterologous cells, but instead of using whole cells or fragments of membrane protein carrying ORs, this purifies the ORs using some specific detergents and reconstitutes ORs into a membrane-like liposome to maintain their functionality [21]. The *E. coli* expression system is most commonly used for the production of ORs due to its popularity and ease to engineer. However, in many cases, the expressed proteins are produced in an insoluble form, and thus an additional refolding step is required to give their native structure, solubility and natural function.

With the advances in biotechnology protein techniques, cell-free protein synthesis (CFPS) is a valuable and promising tool to produce ORs in an efficient and cost-effective way. In one study, human OR (hORI7-4) was produced using a cell-free system and applied as a sensing element to detect odour molecules [59]. Advantages of the use of CFPS include easy modification of reaction conditions to favor protein folding, decreased sensitivity to product toxicity, suitability for high-throughput strategies, easy modification of the specific site that is useful for surface immobilization [60,61]. In CFPS, as a template, an exogenous mRNA or DNA is used to directly synthesize proteins of interest outside the living cells. The CFPS system consists of all the necessary substances, including an exogenous supply of target gene, essential amino acids, nucleotides, buffer solutions, energy-generating factors, cell extracts, etc. [62].

### 3.2. Immobilization of ORs

In a bioelectronic nose system, the functional coupling between sensing element (whole cells, ORs, OBPs) and transducers is critical for the performance of biosensors. The successful immobilization of functional ORs onto a biosensor surface requires efficient capture of ORs and maintains its native functions during the analysis process. Often, ideal immobilization of ORs greatly improves their stability by minimizing protein unfolding [63]. The common methods of OR immobilization for bioelectronic nose systems are outlined in Table 4.

Currently, there are three main and common methods used for the immobilization of sensing element onto transducers, which are: physical adsorption, specific binding by antibodies or binding peptides and covalent immobilization through chemical reactions. Among these, physical adsorption immobilization is quite simple to perform, requiring a solution containing ORs to be evenly coated onto a sensor surface [64]. Due to its simplicity and convenience, this has been applied and developed widely with many effective research results [12,27]. However, physical adsorption undergoes the lack of sufficient binding strength and the low stability. Moreover, in addition to the target OR protein, other contaminant proteins are also capable of binding to the surfaces, which could affect the performance of a biosensor.

To overcome these drawbacks, self-assembled multilayer immobilization using antibodies has been developed in order to improve the specificity and stability of OR-based bioelectronic noses. The use of suitable antibodies that specifically recognize ORs can help immobilize the OR proteins on the sensor surfaces. There are several steps in this antibody-based self-assembled multilayer immobilization. First, a mixture of self-assembled multilayer containing a biotinyl group is formed on a gold surface via Au–S bonds. Next, via biotinyl–neutravidin binding, neutravidin is bound on the surface. After that, biotinylated specific antibodies are deposited onto the substrate, and finally, ORs are specifically captured by immobilized antibodies to form a specific and stable immobilization structure [65]. This technique was used to immobilize ORs on the sensor surface to detect odour molecules using a biosensor based on electrochemical impedance spectroscopy [66]. Another interesting study on self-assembled multilayer immobilization was achieved by Vidic et al., in which antibodies were used for specifically capturing ORs located in nanosomes on a sensor surface [67]. Their method offers advantages such as higher specificity, affinity and stability. More importantly, other irrelevant membrane proteins are washed away, thus minimizing the use of additional purification processes. Even though this approach can overcome the shortcomings of previous techniques, it still has some disadvantages, including the requirement of an additional process for antibody-binding protein immobilization, and unsuitability for sandwich assays.

Recently, a direct covalent immobilization through selective and stable chemical reaction mainly based on Au–S self-assembly process has been reported [68,69]. The covalent immobilization of ORs has the supposed advantage of irreversible binding of the OR proteins to the sensor surfaces. Usually, the nucleophilic functional groups present in amino acid side chains of proteins and groups such as amino, carboxylic, sulfohydryl imidazole, thiol, hydroxyl, phenolic, threonine, indole, etc. are used for covalent coupling [70,71]. Sankaran et al. used this technique to immobilize synthesized ORs onto a gold substrate [60,72]. In their study, ORs containing a cysteine residue on one terminal provided thiols for covalent binding on the gold surface. Zhou et al. reported the development of an amperometric biosensor based on the covalent immobilization of tyrosinase on a boron-doped diamond (BDD) electrode to detect phenolic compounds [73]. In that study, to the modified surface, a carbodiimide coupling reaction was used to covalently immobilize tyrosinase on the BDD surface. In general, in comparison with the case of physical adsorption and self-assembly with the specific antibodies method, covalent binding immobilization is more likely to lead to greater strain on the OR protein due to harsh immobilization conditions. Therefore, covalent immobilization may induce conformational changes of protein structures and active sites to fit the substrate after binding, resulting in loss of activity and alteration of the substrate specificity. However, the binding force between the receptor protein and the sensor surface is so strong that even when exposed to a substrate or solution of high ionic strength, the bound OR proteins hardly leak into the buffer solution.

## 4. Applications of Bioelectronic Nose

**Figure 5:**
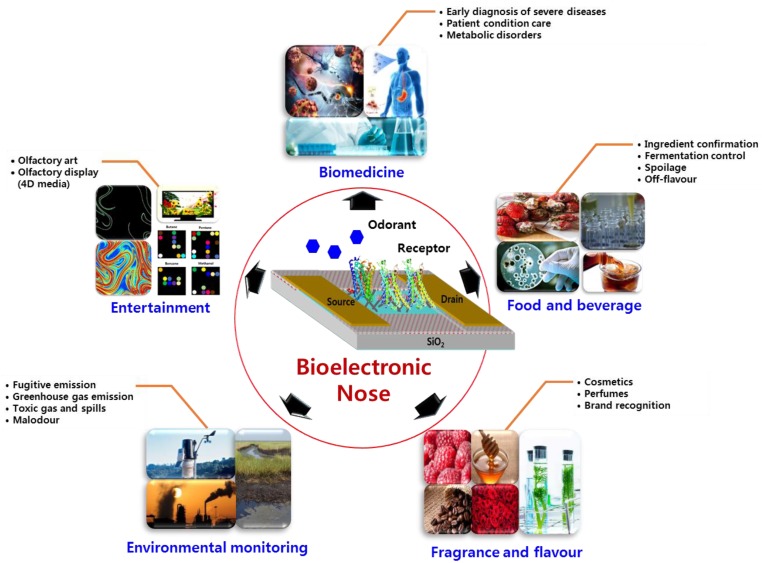
Applications of bioelectronic nose in the areas of biomedicine, food and beverage, fragrance and flavour, environmental monitoring and entertainment.

Investigators have conducted extensive studies in the electronic analysis of odorant molecules using bio-inspired electronic noses [13,14]. Currently, various kinds of bioelectronic noses are being applied for olfactory analysis owing to the unique electrical and biological properties by integrating nano-devices with biological recognition elements, thereby elevating the sensitivity and specificity of detection. In this regard, a bioelectronic nose might be appropriate for applications in various fields requiring this purpose such as food quality control, environmental monitoring and even in medical diagnosis. Figure 5 shows areas of applications of bioelectronic nose, and comparison of OR-based biosensors used for various applications are listed in Table 5.

### 4.1. Applications in Medical Diagnosis

The skin, sputum, urine and breath are disease-correlated odour sources. Previously, only the compositions of human fluids such as the blood and urine were analyzed, but recently, the analysis of human breath also has been accepted as a good diagnostic tool in clinical diagnosis. Chemical compounds from the human body are important indicators that could be used to diagnose various kinds of human diseases as biomarker compounds. In particular, exhaled breath consists of numerous VOCs that can provide information about the physical condition of patients. Unsurprisingly, using smell as an indicator of disease probably originated with the Greek physician Hippocrates around 400 BC [79]. Observations that unusual human odours provided some indication of human ailments helped early medical practitioners to recognize that certain diseases might alter the way a person’s body odour smells [80,81]. These volatile compounds released from the body provide information about health conditions, such as infections, intoxication, or metabolic diseases [82]. The non-invasive diagnosis for various diseases is a great advantage of breath testing over invasive techniques requiring endoscope and biopsy [83]. Thus, VOCs analytical methods such as gas chromatography/mass spectroscopy (GC/MS) [84], which is the most common form of measuring volatile compounds, selected-ion flow-tube mass spectroscopy (SIFT-MS) [85], proton transfer reaction-mass spectrometry (PTR-MS) [86], and semiconductor metal oxide (SMO)-based gas sensors [87] have been widely adopted to detect many types of VOCs at the sub-ppm level in exhaled breath. Electronic noses using biological recognition based on living cells, proteins, or peptides, can be used to detect physiological or biochemical processes with high sensitivity and selectivity. As a pioneering study on bioelectronic nose applied to disease diagnosis, Lin et al. developed an electronic nose integrated with synthesized peptides designed by simulating the three-dimensional structure of the OR docking with volatile molecules for the detection of odorant biomarkers of the uremia [74]. In order to develop a diagnostic breath test, it is necessary to identify disease-specific VOC biomarkers. Various important VOC biomarkers have been determined for the diagnosis of cancer [47]. In particular, for the rapid diagnosis of lung cancer, much effort has been devoted to the investigation of electronic nose systems to analyze the exhaled breath, which is essential to early treatment. In addition, OR-based biosensors can be applied in drug discovery by detecting the interaction between ORs and drugs [88]. The electronic nose, as a reliable, time-saving and economic diagnosis device, has the potential to be practically used in medical applications.

### 4.2. Applications in Food Quality Control

**Figure 6:**
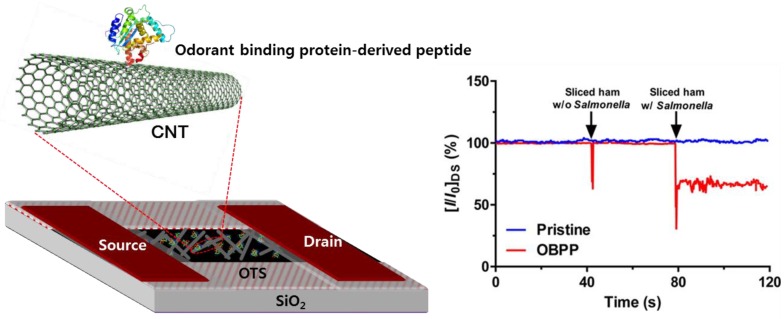
Schematic diagram of a bioelectronic nose using carbon nanotube field-effect transistor functionalized with odorant binding protein-derived peptides (**Left panel**). The peptides were directly immobilized via π–π interactions between Phe residues and CNTs. Real-time detection of *Salmonella* contamination in sliced ham (**Right panel**). Adapted from [75].

Food safety is one of the key issues for maintaining and promoting human health. In this respect, quality control of food is also an essential field that should not be overlooked [89]. Threats to food safety caused by specific pathogenic bacteria have shown that it is imperative to develop systems that can quickly and accurately detect microbial spoilage; therefore, various electronic nose techniques have been applied to proactively inspect and control foods that are very vulnerable to deterioration [59,72]. The olfactory-based biosensor has provided an effective detection method for rapidly, accurately and reproducibly monitoring foodborne pathogens from packaged foods [90]. In one study in 2012, Panigrahi et al. applied a QCM system coated with olfactory receptors to recognize acetic acid, which is associated with *Salmonella* contamination of packaged meat [91]. Synthetic polypeptide molecules were attached on a QCM electrode and the olfactory receptor-based synthetic polypeptide sensor was evaluated for detecting acetic acid in low concentrations at 10–100 ppm and at room temperature. In that study, mean estimated LOD (limit of detection) of the QCM bioelectronic nose was about 2 ± 1 ppm, indicating that OR-based QCM system is applicable for detection of packaged meat spoilage and contamination. The olfactory receptor-based *Salmonella* detection system can contribute greatly to food safety as a technology suitable for rapid detection and primary screening. Recently, Son et al. developed a bioelectronic nose based on *Drosophila* odorant binding protein (OBP)-derived peptide and carbon nanotube field-effect transistor (CNT-FET) for detection of *Salmonella* contamination in ham (Figure 6) [75]. When odour molecules stimulate the olfactory system, OBP, which is a soluble protein in olfactory mucus, binds with the odorant and transfers it to olfactory receptors. The authors demonstrated that the peptide-based bioelectronic nose sensitively detected 3-methyl-1-butanol at a concentration of 1 fM and selectively distinguished the target odour molecule from other compounds with similar structures.

In fruit production, the age of the fruit determines the shelf life and quality loss due to changes in freshness, flavour, firmness and color. Therefore, to ensure the good quality of fresh fruits at the post-harvest stage, it is necessary to harvest fruits at optimal physiological conditions. Recently, it has been demonstrated that odorants emitted by fruits are correlated with fruit maturity and quality, potentially being used as odour markers. Studies have demonstrated that bio-inspired electronic nose technology can be used to monitor the maturity of fruits by showcasing that three maturity indices such as puncture, soluble solids and starch can be measured using bioelectronic noses [46]. The results of PCA and DA analysis clearly showed that a bioelectronic nose could classify gala apples into three maturity groups. In food detection technology, the vapour stage of food-related chemicals is very important, because these could be utilized for target ligands, especially in bioelectronic noses to control the quality of food. The odorant biomarker of food can be used to distinguish food conditions. First of all, in order for a biomarker to be practically usable, the validity of these biomarkers must be ascertained. Once the odour biomarker is validated, the receptor-based electronic nose can be used to quickly detect pathogens in food samples. In cheese production, flavour is closely associated with the ripening process that depends on the growth of bacteria, lipid degradation and oxidation, and proteolysis. Thus, it is not simple to confirm the absence of anomalous smells by the cheese odours. Traditionally, sensory evaluations have been utilized for the determination of the quality and identity of cheese. However, current methods by a specific test relying on expert panels are time-consuming and costly. With regard to perfume, a mixture of fragrant essential oils or aroma compounds are important ingredients in the development of products in the cosmetics and perfume industries. Connoisseurs of perfume become extremely skillful at identifying components and origins of scents. Thus, there is a need for a bioelectronic nose with reliability, short response time and cost-effectiveness in the fragrance and flavour industries.

### 4.3. Applications in Environmental Monitoring

Methods for monitoring most chemical contamination in the environment are costly and time intensive, and they involve limited sampling and complicated sensing techniques. Therefore, the demand for cheap, improved, and reliable methods for rapid, accurate detection and quantification of environmental chemical pollutants are increasing. In these situations, bio-electronic nose technology, which is based on the combination of gas sensor technology with the ability to detect a wide range of organic and inorganic vapour, especially chemical pollutants, and bio-interfacing technology, is the best solution. These devices are based on a variety of operational principles and can be used to control chemical pollution in many environmental applications. They can be widely applied to environmental monitoring of urban pollutant emissions for the purposes of air pollution monitoring, early or real-time area monitoring via sensor monitoring networks [92], mapping of chemical plume dispersion to detect fires at chemical-storage facilities [93] and maintaining chemical security at harbor entrances or importation ports [94]. The bioelectronic nose can quickly detect leaks of toxic or hazardous substances in pipelines or industrial plants as well as can potentially alert the premises of the accumulation of organic solvents or explosive fumes such as carbon monoxide or carbon dioxide. Also, this technology is applicable to on-site monitoring of soil contamination, which is recognized as one of the major soil threats.

In environmental applications, metal oxide semiconductor sensors are among the most widely used transducers for on-site monitoring of environmental pollutions due to their characteristics of light weight, cost-effectiveness and robustness [95]. These sensors also have a relatively long-life span and can be reused with fast response and fast recovery time. When it comes to its multiplexibility, the bioelectronic nose allows for multiplex analyses of various contaminants by means of using a gas sensor array with cognate sensing materials for different detection targets [13,96]. Recently, Son et al. developed a bioelectronic nose constructed with hOR and SWNT-FET for the real-time assessment of water quality, showing that the detection limit of the bioelectronic nose was at a sufficiently low level for the detection of geosmin (GSM) and 2-methylisoborneol (MIB) in water [76]. Another approach for environmental analysis was performed using biomimetic polydiacetylene-coated CNT-FET [49]. The SWNT-FET sensor device interfaced with PDA-based lipid membranes coupled with TNT receptors and was exploited to transduce the binding activities between the target TNT and its selective peptide receptors.

### 4.4. Applications in Smell Visualization and Standardization

Nowadays, smell visualization has become a popular and fast-growing field of artificial olfaction. So far, well-trained perfumers and heavy weight analyzers with big sizes such as GC-MS and electronic noses have played a key role in objectively recognizing and distinguishing odorant molecules [97]. Many attempts have been made for smell visualization to objectively express the smell, along the increasing need for classification and codification of odours. To date, various methods such as calcium imaging [98], cAMP response element (CRE) reporter assay [99] and bioluminescence resonance energy transfer (BRET) [100] based on olfactory cells engineered have been developed for smell visualization. However, these methods have disadvantages in that it is difficult to consistently control the cell state and it is impossible to detect low concentration of odorants. In order to realize visualization of odours, a colorimetric sensor array acting as an optoelectronic nose has been developed, but still has limitations on its sensitivity to smells and the number of olfactory stimuli that can be visualized [101]. Recently, studies have demonstrated that a bioelectronic nose that mimics human olfactory system can be applicable for implementing smell visualization [78,102]. Once a bioelectronic nose that integrates all human olfactory receptors into a single chip is developed, the system can potentially detect all the possible smells that humans can recognize. For successful smell visualization, it is required that the response of engineered olfactory cells to olfactory stimuli should be converted into visual images using various methods to measure the intracellular signals [97]. In forthcoming years, the development of smell visualization devices based on bioelectronic nose is expected to enable anosmic patients to perceive smells that have not been sensed before. Furthermore, with regard to sensory rehabilitation, recent report by, Liu et al. has shown that a flexible circuit was successfully injected into the living brain [103]. The mesh electronics injected into the mouse brain exhibited little immunogenicity, attractive interactions with neurons, and can reliably monitor brain activity. With the use of brain computer interface (BCI) technology based on flexible electronics, the realization of the olfactory rehabilitation of people who has smell disorders is expected in the future.

More recently, Son et al. proposed a concept for odour standardization using an OR-based bioelectronic nose that encodes odour information and identifies primary odorant molecules [104]. Conventional approaches to odour standardization include sensory evaluation of smell based on scoring by expert panels and electronic nose measurement based on chemical sensor arrays. However, traditional electronic nose has shortcoming in expressing a large number of odours. The authors mentioned that the following development is required for smell standardization. 1. Standardization of bioelectronic nose devices for odour measurement; 2. Coding of specific odours; 3. Selection of standard primary odorant molecules which can play a role similar to the three primary colors of light; 4. Establishment of odour classification system. Once smell standardization is successfully achieved, the bioelectronic nose with multi-channel sensing arrays can offer a variety of odour information using pattern recognition technology, and the odour can be even reproduced through the integrated olfactory display system. 

## 5. Conclusions

In this review, we have presented and evaluated the most recent progress in olfactory receptor-based biosensors. Due to their significant advantages of high sensitivity and specificity based on the natural binding of ORs to their specific ligands, OR-based biosensors hold the most potential to be employed in bio-inspired electronic nose sensor systems for recognizing VOCs in many fields including clinical diagnosis, food safety, environmental and industrial monitoring, and agriculture. With increasing understanding of ORs and OBPs, synthetic proteins and peptides are increasingly being used as substitutes for tissues and cells for the recognition of specific odorants [34,75]. It is noteworthy that much attention has been paid to ionic liquids (ILs)-based electrolytes over the past decade. ILs have been applied to various fields such as batteries, capacitors, nonvolatile memory devices, biosensors, etc. due to their attractive characteristics, which include low vapour pressure, high capacitance, and excellent thermal, chemical and electrochemical stability [105,106]. Recently, FET-type devices and logic circuits that operate at voltages as low as a few voltages were fabricated using ILs as gate insulators of electrochemical transistors [107,108]. Along this line, the bioelectronic noses using ILs are expected to be intensively and extensively studied and developed as an important element of the next generation of odour biosensors including flexible and wearable electronic devices. Furthermore, trained dogs, rats, bees and *Drosophila* have been used to detect drugs or explosives, or even in medical applications. Studies on the olfactory responses of these trained animals have helped to developed bio-inspired electronic noses that can turn up all over the place to speed up testing [109]. Despite its promising prospects, in practice, the OR-based biosensor is an early-stage technology, and so far no commercialized bioelectronic nose has been marketed. When it comes to the commercial availability of bioelectronic nose systems, some issues such as stability of biomolecules, reproducibility, cost-effectiveness and response speed still remain to be resolved before the practical application of the bioelectronic nose can be implemented. Among these, the stability of bioelectronic noses is one of the most significant drawbacks in their variety of applications. Recently, some interesting studies on the stability of OR-based bioelectronic noses have reported. Park et al. examined the lifetimes of graphene-based FET-type bioelectronic noses conjugated with human ORs by storing them in a sealed vessel, reporting that OR-based graphene FET showed excellent stability (95% of the activity was maintained at room temperature after 10 days) [14]. Another study by Lee et al. has shown that the sensitivity of carboxylated polypyrrole nanotubes (CPNTs) functionalized with human ORs maintained more than approximately 60% funtionality, when the bioelectronic nose was stored at 25 °C for 10 weeks in air-dried conditions [11]. In addition, the need to detect gaseous odorant molecules is another critical issue of this bio-inspired nose system. The ORs exist in an aqueous environment, yet detecting odorants that are primarily hydrophobic and well-vapourized [110]. If only ORs remain active in dry conditions, the limitation can be solved. Given that only the wet protein samples are functionally active, it is challenging to keep the ORs constitutively active under dry conditions. As a promising candidate to figure out this problem, nanodiscs can be regarded because they possess the ability to provide a native-like environment to membrane proteins and thus binding of odorants onto ORs can occur in a near physiological state [19,111]. Along with advances in nanotechnology, the integration of ORs with nanostructured devices enables the bioelectronic nose to have high sensitivity and specificity. The powerful innate detecting capacity of the biological olfactory system may offer great insights into biomimetic odorant sensors with high performance, thereby improving the ability to identify as well as to discriminate odorants in complex environments. The functions of all ORs have not been understood yet. Following further investigation of their structural and functional properties, the application area of bioelectronic noses will continue to grow. Therefore, in the near future, OR-based biosensors will undoubtedly reach the level of commercialization, and show promising prospects in various applications. We believe that bioelectronic noses can be useful for applications in various field such as food monitoring, law enforcement, homeland security, environmental monitoring, diagnostic breath testing, etc.

## Figures and Tables

**Table 1:** A brief history of bioelectronic noses.

Year	Inventor	Object of the Invention	Ref.
1998	Gopel et al.	Concept of bioelectronic nose	[22]
1999	Wu	A piezoelectric electrode used in the immobilization of a crude bullfrog cilia as a signal transducer	[27]
2006	Lee	SPR system to characterize molecular interaction between olfactory receptor and its cognate odour molecule	[29]
2005	Ko & Park	Whole cell-based QCM sensor system for selective recognition of odorant molecules	[32]
2006	Sung et al.	A crude membrane expressing an olfactory protein was used for measuring odorants using a quartz crystal microbalance (QCM)	[28]
2011	Goldsmith et al.	Biomimetic chemical sensors using nanoelectronic read out of olfactory receptor proteins	[19]
2012	Park et al.	Ultrasensitive flexible graphene based field-effect transistor (FET)-type bioelectronic nose	[14]
2012	Jin et al.	Nanovesicle-based bioelectronic nose platform mimicking human olfactory signal transduction	[33]
2013	Lim et al.	Peptide receptor-based bioelectronic nose for the real-time measurement	[34]
2014	Oh et al.	Odorant detection using liposome containing olfactory receptor in the SPR system	[21]
2014	Lu et al.	Olfactory biosensor using odorant-binding proteins from honeybee	[35]
2015	Di et al.	A surface acoustic wave bioelectronic nose for detection of volatile odorant molecules	[20]

**Table 2:** Examples of ORs-based bioelectronic noses. Adapted from [48].

Sensor Type	Analytes	Sensitivity	Ref.
Olfactory receptor proteins(ORPs) from bullfrogs (Rana spp.) coated onto the surface of a piezoelectric (PZ) electrode	*n*-caproic acid, isoamyl acetate, *n*-decyl alcohol, linalool, ethyl caporate	10^−6^–10^−7^ g	[27]
Quartz crystal microbalance (QCM) was coated with ODR-10 receptor (*C. elegans*)	Diacetyl	-	[28]
hOR 2AG1 (hOR2AG1) conjugated carboxylated polypyrrole nanotubes (CPNTs) field-effect transistors (FETs)	Amyl butyrate	10 fM	[44]
Trinitrotoluene (TNT) receptors bound to conjugated polydiacetylene (PDA) polymers with single-walled carbon nanotube field-effect transistors (SWNTFET)	Trinitrotoluene	1 fM	[49]
A liquid-ion gated FET B-nose using human olfactory receptors 2AG1 (hOR2AG1: OR)-conjugated modified bilayer graphene (MBLG)	Amyl butyrate	0.04 fM	[14]
An olfactory-nanovesicle-fused carbon-nanotube-transistor biosensor(OCB) with canine ORs(cfOR5269)	Hexanal	1 fM	[46]
Single-walled carbon nanotube-based FETs (SWNT-FETs) with human OR 2AG1 (hOR2AG1)	Amyl butyrate	1 fM	[33]
Single walled-carbon nanotube field-effect transistors (SWNT-FETs) functionalized with olfactory receptor-derived peptides (ORPs)	Trimethylamine	10 fM	[34]
Nanovesicle-based bioelectronic nose (NvBN) with 30 types of human olfactory receptors (hORs)	Heptanal	10 fM	[47]
Multiplexed superbioelectronic nose (MSB-nose) using graphene micropatterns (GMs) and field-effect transistor (FET) with two different hORs (hOR2AG1 and hOR3A1)	Amyl butylate, helional	0.1 fM	[13]
Olfactory receptor-derived peptides(ORP)-coated Single-walled carbon nanotube-field effect transistors (SWNT-FETs) based on a novel microfluidic system (μBN)	Trimethylamine	10 ppt	[30]
An array of five Surface acoustic wave (SAW) resonators coated with three types of odorant-binding proteins (OBPs): the wild-type OBP from bovine (wtbOBP), a double-mutant of the OBP from bovine (dmbOBP), the wild-type OBP from pig (wtpOBP)	R-(–)-1-octen-3-ol (octenol), R-(–)-carvone (carvone)	0.48 ppm 0.74 ppm	[20]
Human olfactory receptor (OR) nanovesicle integrated single-walled carbon nanotubes field-effect transistors (SWNT-FETs)	1-octen-3-ol	1 fM	[31]
Zinc Nanoparticles (NanoZn) equipped biosensor based on olfactory receptor cells bombined with Zinc Nanoparticles (MEA)	Isoamyl acetate, acetic acid	10^−15^ M	[50]

**Table 3:** Summary of OR production methods for OR-based biosensors. Adapted from [24].

Methods	Advantages	Disadvantages
Extracts from tissue or cells	Native structures and functions, native intracellular connections, suitable for physical absorption	Poor reproducible isolation and reconstitution yield of ORs, hard to purify specific ORs, strict storage requirements, need to kill animals
Cell-based expression	Nature membrane for ORs, Grafting of tags, single type of ORs	Low expression efficiency, relatively expensive, time consuming
Cell-free production	High efficiency and purity, controllable reaction conditions	High technique-demanding, relatively high cost
Chemical synthesis	Stable secondary structure, low cost and high purity, site-specific modification	Limited by yields in the range of about 70 amino acids, hard to maintain domains, depend on right sequences

**Table 4:** Examples of OR immobilization methods for OR-based biosensors.

Methods	Advantages	Disadvantages
Physical adsorption	Regent-free/low cost, simple to perform, non-destructive toward ORs	Insufficient binding strength, nonspecific adsorption, low stability
Self-assembly with specific antibodies	Higher specificity/affinity, higher stability, minimizing additional purification processes	unsuitability for sandwich assays, additional process for antibody immobilization
Covalent binding	Strong/irreversible binding force, high uniformity, controlled immobilization	Longer incubation time, conformational changes, loss of ligand specificity

**Table 5:** Comparison of OR-based biosensors used for various applications.

Application Fields	Transducer Type	OR Type	Immobilization Methods	Analytes	Sensitivity	Ref.
Medical diagnosis	SWNT-FET	HEK-293 cells expressing hORs	Self-assembly of CNT-vesicles	Heptanal	10 fM	[47]
	Quartz crystals array	ORs docking with odorants-simulating synthetic peptide	-	Trimethylamine, Dimethylamine, Monomethylamine, Ammonia	Accuracy 86.78%	[74]
Food quality control	QCM	OBP-derived synthetic peptide for alcohol binding	Au–S bonding	Alcohol	<5 ppm	[72]
	CNT-FET	OBP-derived synthetic peptide for alcohol binding	π–π stacking interactions	3-methyl-1-butanol	1 fM	[75]
Environmental monitoring	SWNT-FET	Peptide receptor-PDA vesicles	Self-assembly of CNT-vesicles	Trinitrotoluene	1 fM	[49]
	SWNT-FET	Nanovesicles carrying hOR51S1, hOR3A4	Self-assembly of CNT-vesicles	Geosmin, 2-methylisoborneol	10 ng·L^−1^ 10 ng·L^−1^	[76]
Smell visualization	PEG microwell-based CRE reporter assay	HEK-293 cells expressing hORs	-	Helional	50 nM	[77]
	Fluorescence image scanning	HEK-293 cells expressing ion channel-fused hORs	-	Amyl butyrate	2 nM	[78]
